# Rare case of left anterior descending artery compression

**DOI:** 10.1093/ehjcr/ytag018

**Published:** 2026-01-23

**Authors:** Lina Kapleriene, Dominykas Sudavicius, Diana Zakarkaite, Sigita Glaveckaite

**Affiliations:** Clinic of Cardiovascular Diseases, Institute of Clinical Medicine, Faculty of Medicine, Vilnius University, Santariskiu st. 2, Vilnius 08406, Lithuania; Clinic of Cardiovascular Diseases, Institute of Clinical Medicine, Faculty of Medicine, Vilnius University, Santariskiu st. 2, Vilnius 08406, Lithuania; Clinic of Cardiovascular Diseases, Institute of Clinical Medicine, Faculty of Medicine, Vilnius University, Santariskiu st. 2, Vilnius 08406, Lithuania; Clinic of Cardiovascular Diseases, Institute of Clinical Medicine, Faculty of Medicine, Vilnius University, Santariskiu st. 2, Vilnius 08406, Lithuania

## Case description

**Figure 1:**
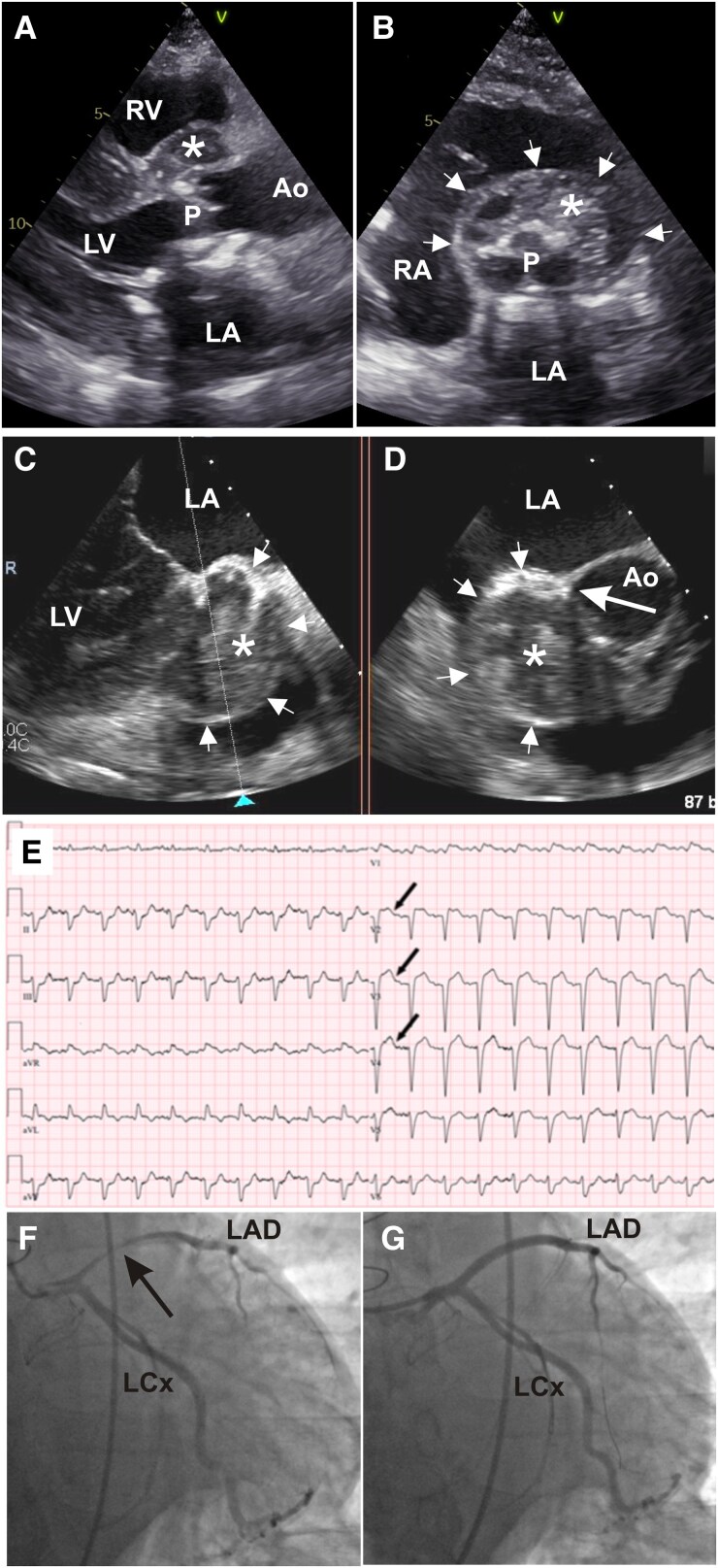
Transthoracic echocardiogram parasternal long-axis (*A*) and short-axis (*B*) views with an asterisk representing the abscess and arrows marking its borders. Transoesophageal echocardiogram two-chamber view (*C*) and a view perpendicular to the former (*D*) show the abscess (asterisk) and its boundaries (small arrows). The left coronary artery’s ostium is indicated by the big arrow (*D*). Electrocardiogram showing ST elevations in leads V1–V5 (*E*). Coronary angiogram indicating left anterior descending narrowing before (*F*, arrow) and after stenting (*G*). Ao, aorta; LA, left atrium; LCx, left circumflex artery; LV, left ventricle; P, aortic valve prosthesis; RA, right atrium; RV, right ventricle.

A 64-year-old male patient presented with new-onset chest pain, fever, fatigue, and systolic murmur at the aortic valve (AV) auscultation point. His history revealed a bio-prosthetic AV implantation due to infective endocarditis 6 years ago. Upon admission, the patient was stable with elevated inflammatory biomarkers. Transthoracic echocardiogram showed an aortic root abscess (*Figure 1*A and B; Supplementary material online, *Videos S1* and *S2*). Aortic valve peak ejection velocity measured at 3.86 m/s and mean pressure gradient at 37.63 mmHg. Transoesophageal echocardiogram showed AV destruction with a mass attached to the right cusp of the bio-prosthetic AV leaning into the left ventricular outflow tract (LVOT) (see Supplementary material online, *Video S3*) and an encapsulated 4.1 × 3.4 cm aortic root abscess (*Figure 1*C and D; Supplementary material online, *Video S4*) likely incorporating the proximal part of the left main coronary artery. Two sets of blood cultures as well as a urine culture were obtained and came back negative. The patient was commenced on intravenous empiric antibiotic therapy of ampicillin, oxacillin, and gentamicin. The patient remained stable and continued undergoing additional pre-surgical evaluations as directed by the cardiac surgeon. However, on the fifth day of hospitalization—just before the planned urgent cardiac surgery—the patient’s haemodynamic condition suddenly deteriorated, and the electrocardiogram showed an acute anterior ST-elevation myocardial infarction (*Figure 1E*). Emergency coronary angiography revealed high-grade proximal left anterior descending (LAD) artery stenosis due to extrinsic compression (*Figure 1*F; Supplementary material online, *Video S5*), in addition to the patient’s significant LVOT stenosis. Despite percutaneous coronary intervention with stenting (*Figure 1*G; Supplementary material online, *Video S6*), worsening haemodynamic condition culminated in death. Because the patient was haemodynamically stable, surgery was scheduled on an urgent rather than an emergent basis. However, an earlier surgical intervention might have saved his life. Though a few similar cases that have been described,^1–3^ our case presents a rare and excellent example of multimodality imaging that allows readers to understand the pathophysiology of external coronary artery compression caused by an aortic root abscess.

## Supplementary Material

ytag018_Supplementary_Data

## Data Availability

No new data was generated or analysed in support of this research.

## Notes

**Consent:** ‘Despite the best efforts of the authors, consent for publication was not obtained for this case. Every effort has been made to anonymize the case. The situation has been discussed with the editor.’
