# Supplementary figures and images for: Rare case of left anterior descending artery compression
Source: Eur Heart J Case Rep. 2026 Jan 23;10(2):ytag018. doi: 10.1093/ehjcr/ytag018 (PMC12884406; doi:10.1093/ehjcr/ytag018)

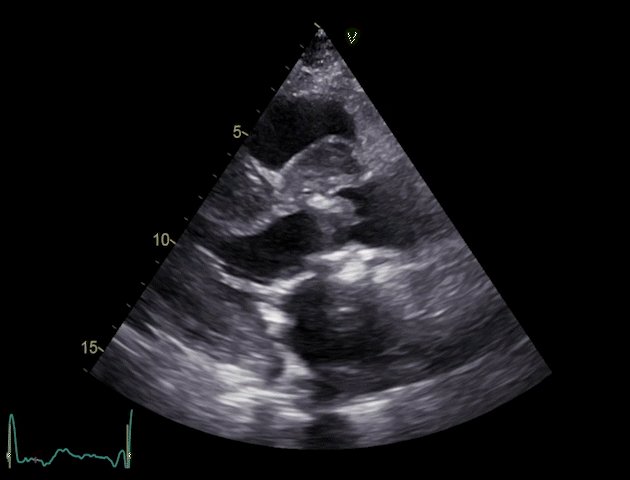

Supplement: ytag018_Supplementary_Data [file ytag018_supplementary_data.zip › Video S1 New.gif]

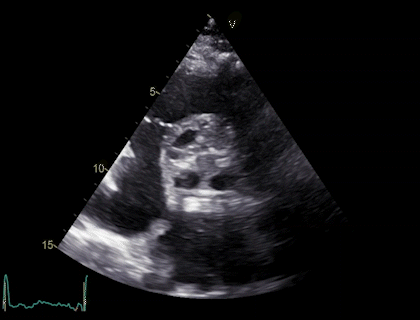

Supplement: ytag018_Supplementary_Data [file ytag018_supplementary_data.zip › Video S2 New.gif]

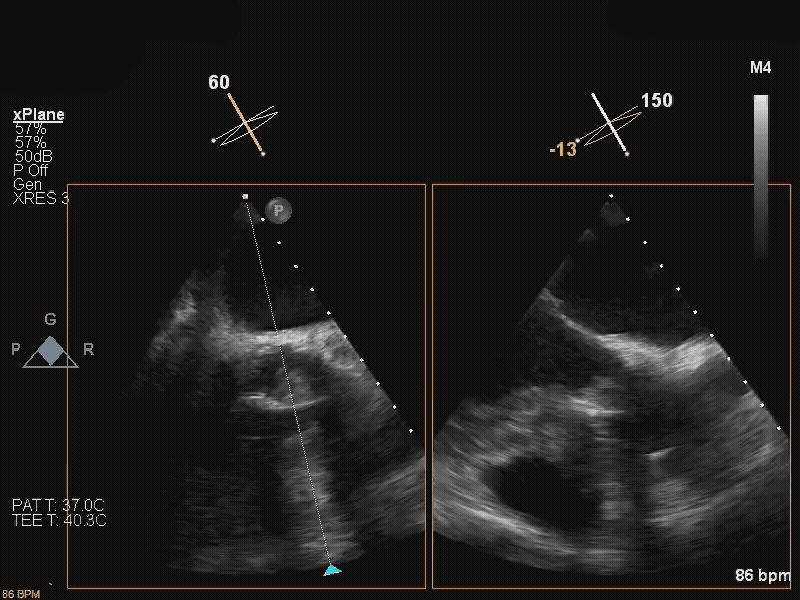

Supplement: ytag018_Supplementary_Data [file ytag018_supplementary_data.zip › Video S3 New.gif]

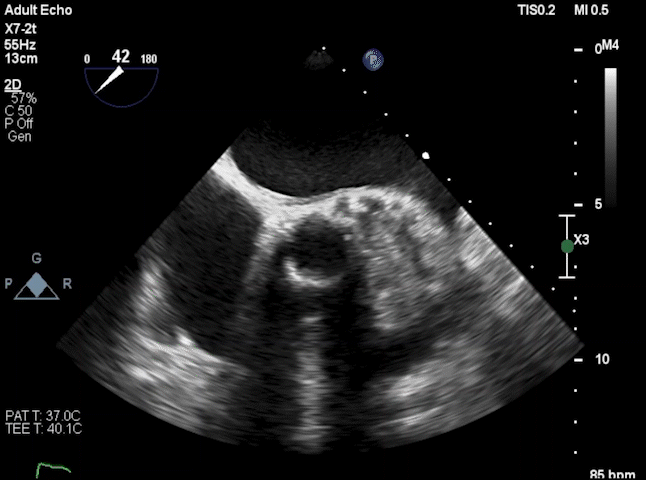

Supplement: ytag018_Supplementary_Data [file ytag018_supplementary_data.zip › Video S4 New.gif]

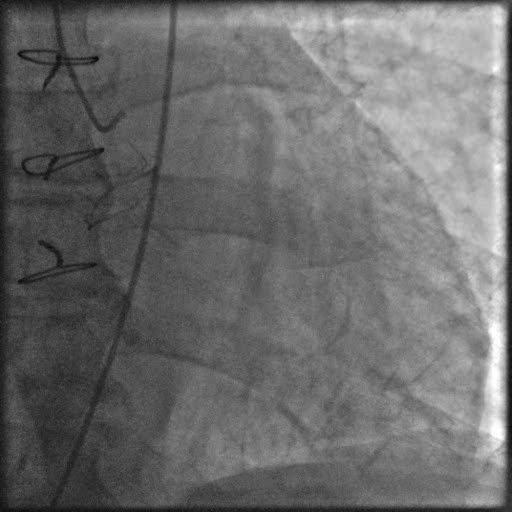

Supplement: ytag018_Supplementary_Data [file ytag018_supplementary_data.zip › Video S5 New.gif]

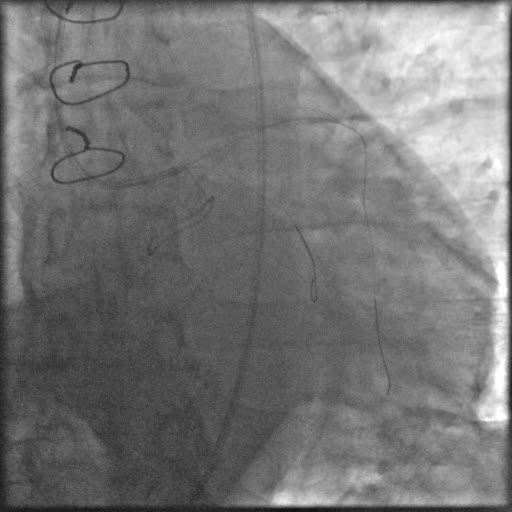

Supplement: ytag018_Supplementary_Data [file ytag018_supplementary_data.zip › Video S6 New.gif]
